# Repeated Autoamputation of the Penis and Successful Replantation for the Second Time: A Case Report and Review of Literature

**DOI:** 10.7759/cureus.101147

**Published:** 2026-01-09

**Authors:** Sebat Karamürsel, Burak Doğan

**Affiliations:** 1 Plastic and Reconstructive Surgery, Ankara Etlik City Hospital, Ankara, TUR

**Keywords:** autoamputation, klingsor syndrome, penile amputation, replantation, secondary amputation

## Abstract

Penile autoamputation is a rare surgical emergency and should be managed carefully to obtain satisfying functional and aesthetic results. Although non-microsurgical techniques were described for penile replantation, microsurgical techniques are still the gold standard treatment to provide the micturition function, return of sensations, and erectile functions. However, the second penile amputation can be difficult to achieve satisfying functional results.

Here, we present a case of a paranoid schizophrenic who performed self-amputation of his penis with a jackknife twice in an interval of two years. The penis was replanted with a microsurgical technique successfully. Minimal ventral skin necrosis was treated with skin grafting at 15th postoperative day. At one-year follow-up, he reported restored urination. Aesthetic and functional results were satisfactory.

Although there is a question of whether it is worth doing the second-time replant in psychiatric patients with self-mutilation injury, the amputation of the penis is associated with psychological and functional implications. Therefore, secondary amputation of the penis should be replanted.

## Introduction

Most cases of penile amputation in adults are caused by self-mutilation, particularly in patients with underlying psychiatric disorders [1,2]. Penile amputation represents a devastating injury with profound functional, psychological, and reconstructive implications, affecting urinary function, sexual identity, body image, and overall quality of life. Other causes include circumcision-related complications, accidental trauma, and assault by spouses or other individuals as an act of punishment or retaliation.

Since the first successful microsurgical replantation of an amputated penis reported in 1977, numerous cases have been described in the literature [3,4]. Microsurgical replantation using an operating microscope has become the current standard of care, as it enables the precise restoration of vascular and neural continuity and offers superior functional outcomes. The primary goals of penile replantation are the preservation of urinary function, followed by the restoration of penile sensation and erectile function [5].

Genital self-mutilation is most commonly associated with psychotic disorders, particularly schizophrenia, where delusions, hallucinations, or religious and sexual guilt may drive self-injurious behavior. Despite the growing number of reported penile replantation cases, repeated self-mutilation, followed by successive penile replantation, remains exceedingly rare. To date, only two such cases have been reported in the literature [6,7]. In this case report, we present a successful second microsurgical penile replantation following repeated self-mutilation in a patient with schizophrenia.

## Case presentation

A 41-year-old male patient with paranoid schizophrenia had a history of penile self-mutilation and successful microsurgical penile replantation performed at another hospital in May 2022. After the initial procedure, the patient was noncompliant with oral antipsychotic medication, and psychiatric follow-up was irregular. He subsequently experienced recurrent auditory hallucinations, which led to a second episode of genital self-mutilation.

In May 2024, the patient again amputated his penis using a jackknife at the level of the mons pubis, several millimeters proximal to the previous amputation line. The injury was caused by a sharp object and resulted in a clean-cut amputation without a crush component. Active bleeding was present at the amputation site and was initially managed with a simple dressing and compression at a local hospital. The patient remained hemodynamically stable, with no signs of hypovolemic shock.

The amputated segment was placed by the patient’s relatives in a clean plastic bag with ice, avoiding direct contact between the tissue and ice. No gross contamination was observed. The patient arrived at our center approximately four hours after amputation, corresponding to the total ischemia time. A distinct warm ischemia period could not be reliably determined.

**Figure 1:**
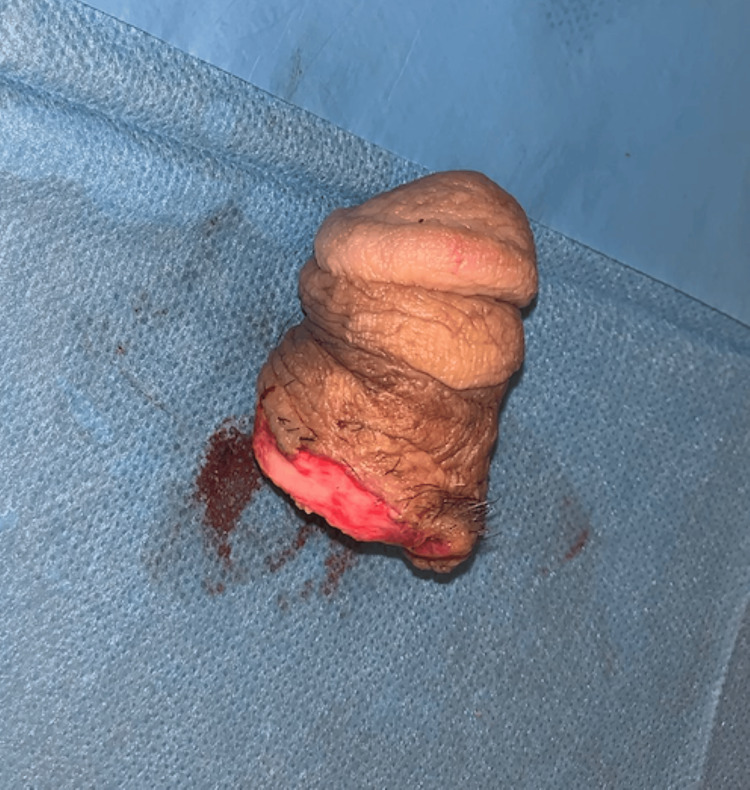
Gross appearance of the amputated penile segment showing a clean-cut amputation with viable skin.

**Figure 2:**
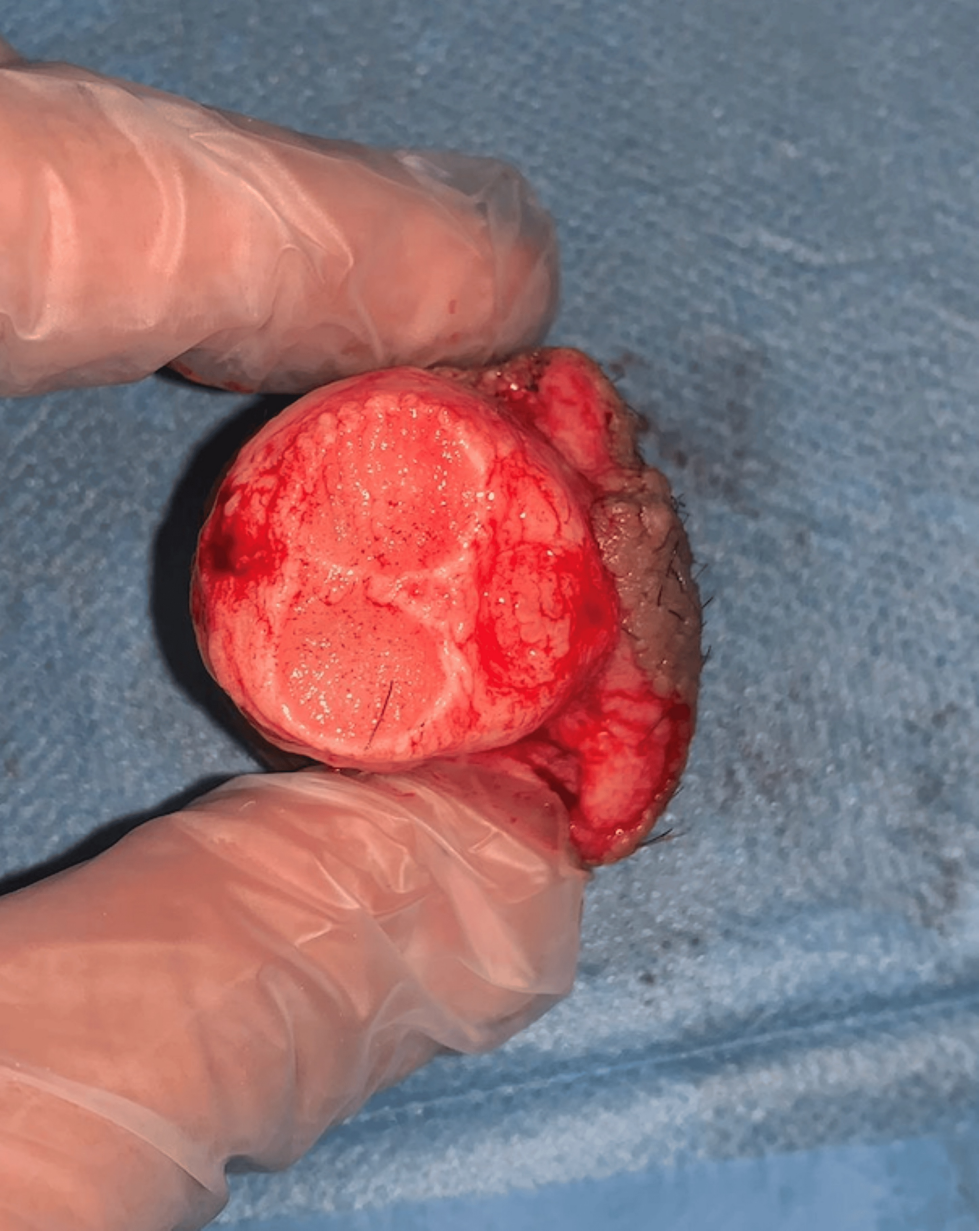
Inferior view of the amputated penile segment containing the urethra, corpora cavernosa, and corpus spongiosum.

The amputated segment was examined under an operating microscope with 4.0× magnification (Figures 1, 2). Both dorsal arteries, the superficial and deep dorsal veins, and both dorsal nerves were identified and found suitable for repair. The cavernosal arteries were unsuitable for anastomosis. The skin of the amputated segment appeared viable, with minimal discoloration at presentation.

**Figure 3:**
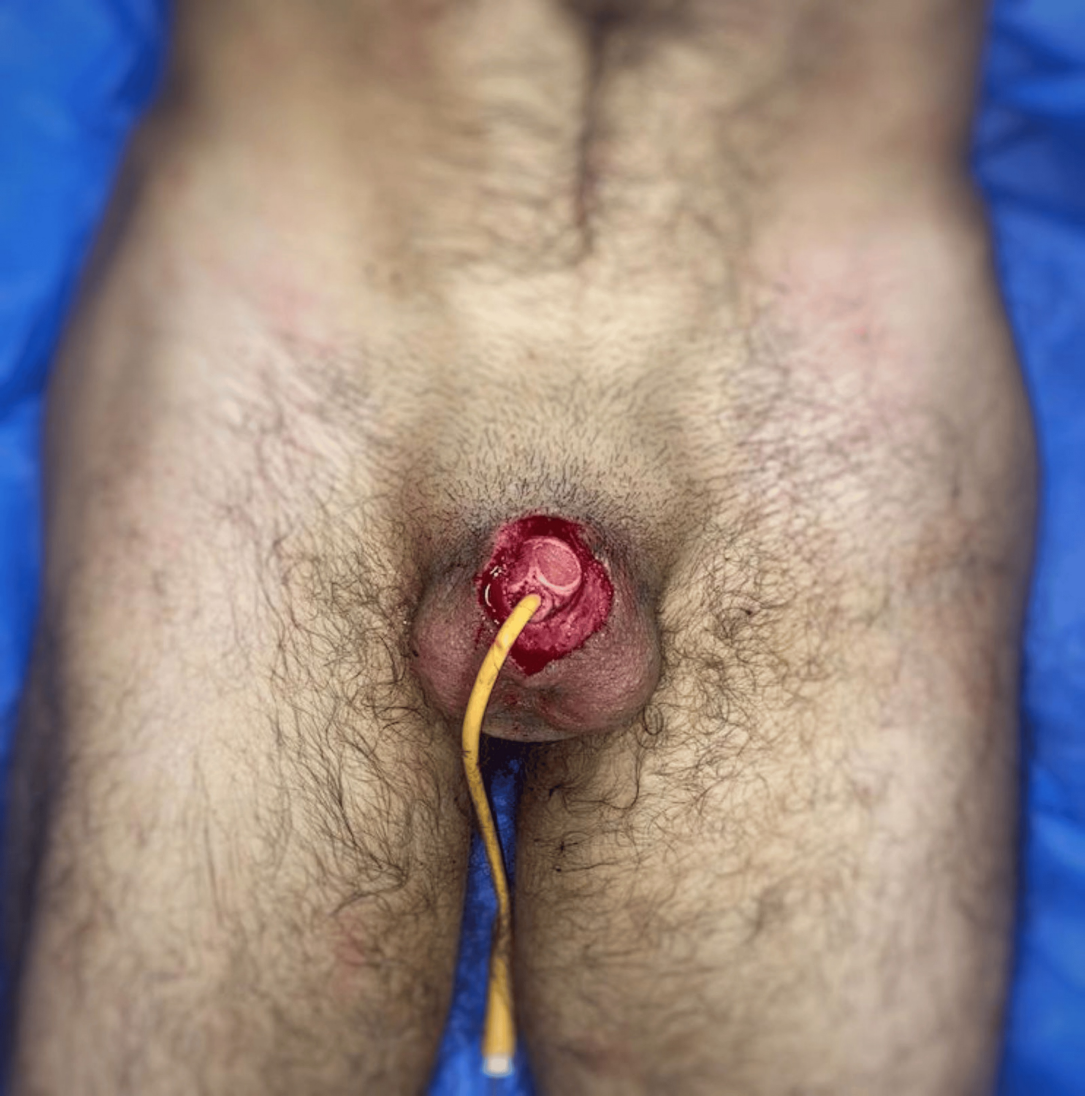
Placement of a 16 Fr Foley catheter through the amputated penile segment and the penile stump to align the urethra prior to microsurgical replantation.

Surgical replantation was performed with the patient in the supine position under general anesthesia. A 16 Fr Foley catheter was passed through the amputated segment and then through the penile stump to align the urethra (Figure 3). The urethra, together with the corpus spongiosum, was anastomosed end to end using interrupted 5-0 round-bodied polydioxanone (PDS) sutures, ensuring a watertight closure. The tunica albuginea of both corpora cavernosa was repaired using 5-0 round-bodied PDS sutures, beginning ventrally and proceeding circumferentially toward the dorsal aspect.

Under microscopic magnification, both dorsal arteries were dissected and anastomosed end to end using 10-0 polypropylene sutures. The deep dorsal vein was reconstructed using a 15 mm interposition vein graft harvested from the dorsum of the foot and anastomosed with 10-0 polypropylene sutures. The superficial dorsal vein was anastomosed primarily with 10-0 polypropylene sutures. Both dorsal nerves were coapted using 10-0 polypropylene sutures.

Urinary diversion was achieved by the placement of a 12 Fr suprapubic catheter in addition to the urethral Foley catheter. The dartos fascia was closed with 4-0 polyglactin sutures, leaving the vascular anastomosis sites tension-free, and the skin was closed circumferentially. The total operation time was six hours.

**Figure 4:**
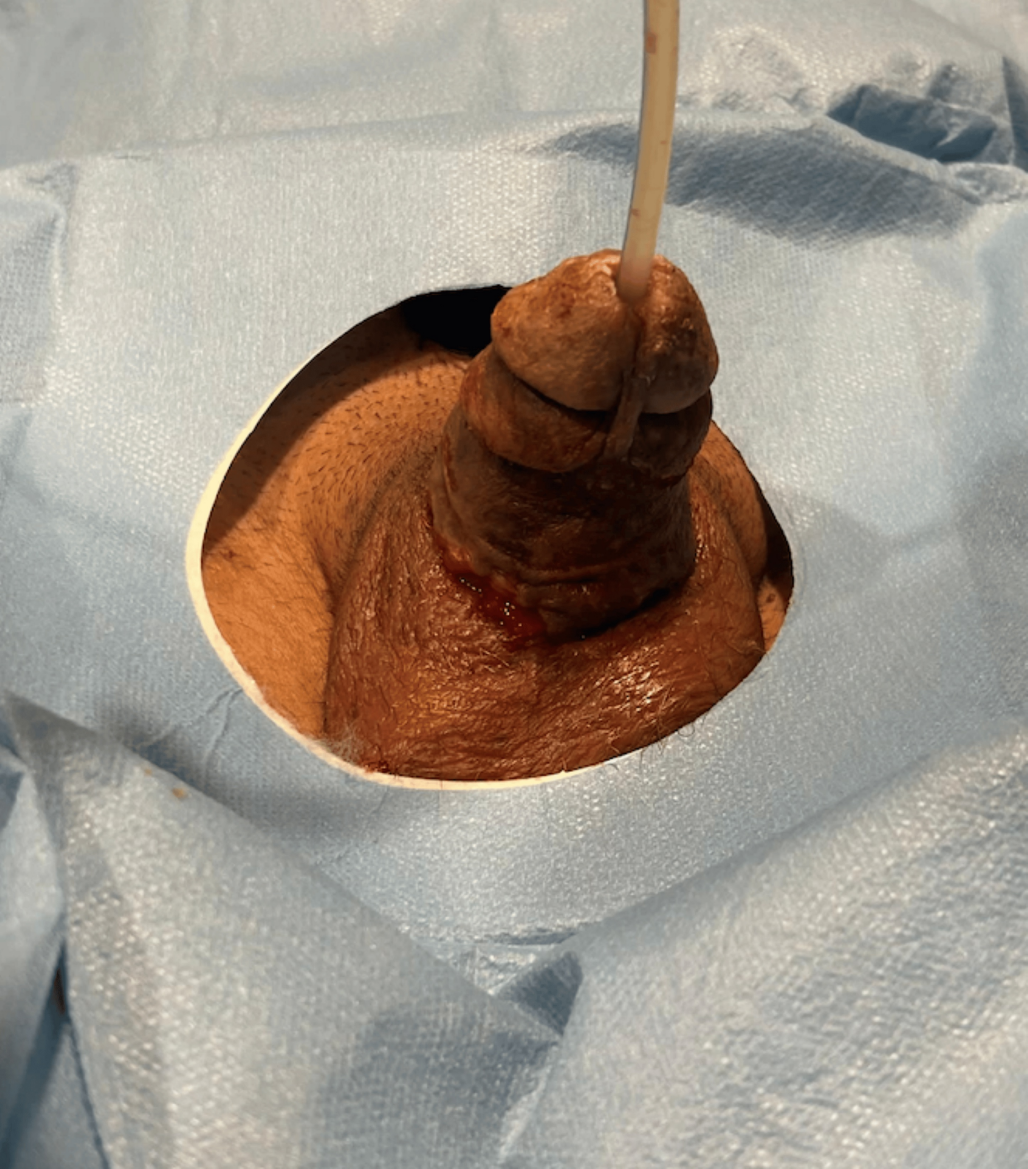
Postoperative first month.

**Figure 5:**
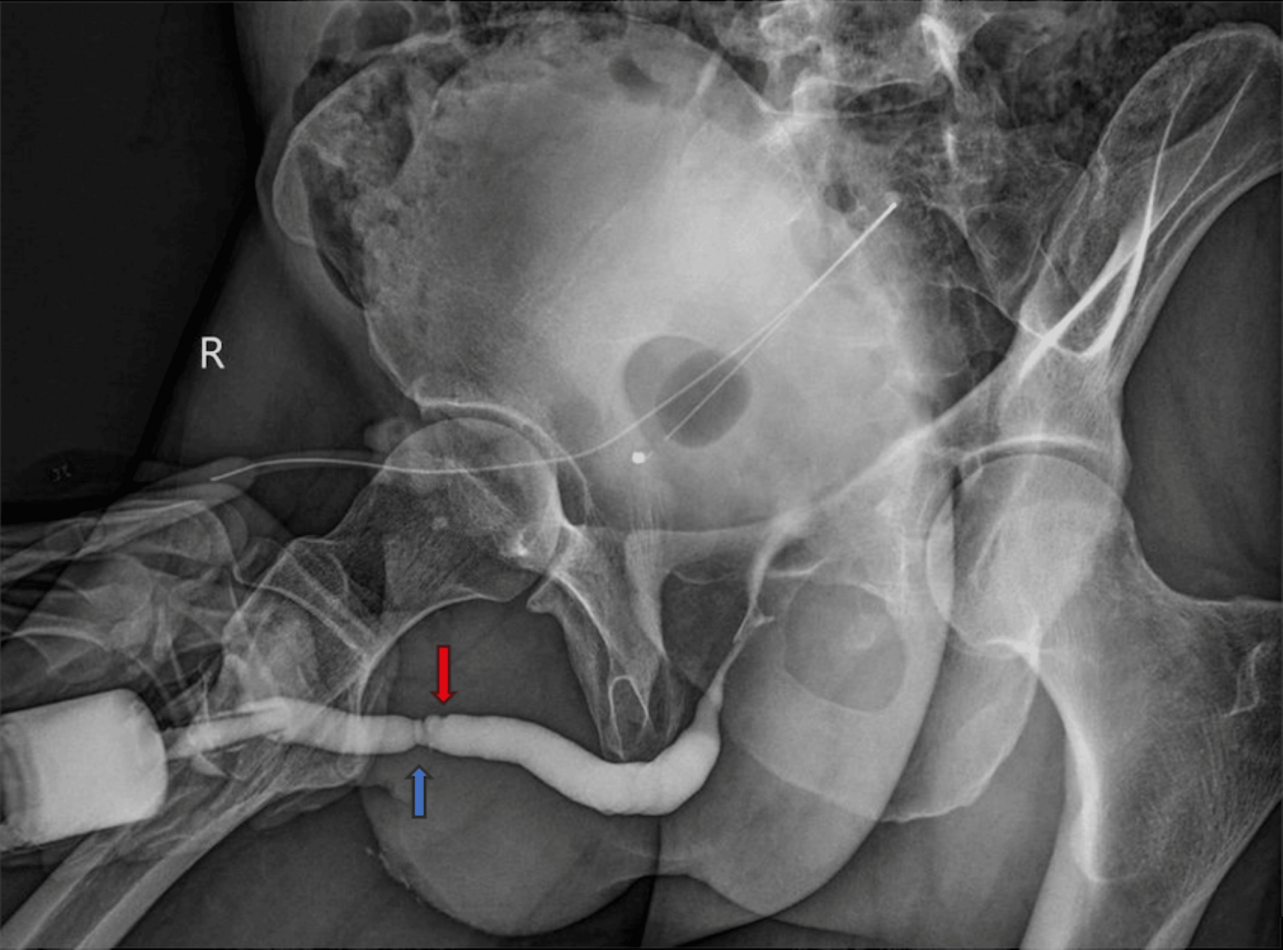
Retrograde urethrography showing the urethral anastomoses; the red arrow indicates the recent and the blue arrow the previous anastomosis.

On postoperative day 14, partial wound dehiscence with minimal skin necrosis was observed on the ventral aspect of the penile skin closure. The necrotic tissue was debrided and resutured. Complete healing was achieved by one month, as demonstrated in the clinical photographs (Figure 4). On postoperative day 30, the urethral Foley catheter was removed, and the suprapubic cystostomy was closed after confirming spontaneous voiding. Retrograde urethrography demonstrated an intact urethral anastomosis without evidence of leakage or stricture (Figure 5). The proximal red arrow indicates the recent anastomosis, and the distal blue arrow indicates the previous anastomosis.

**Figure 6:**
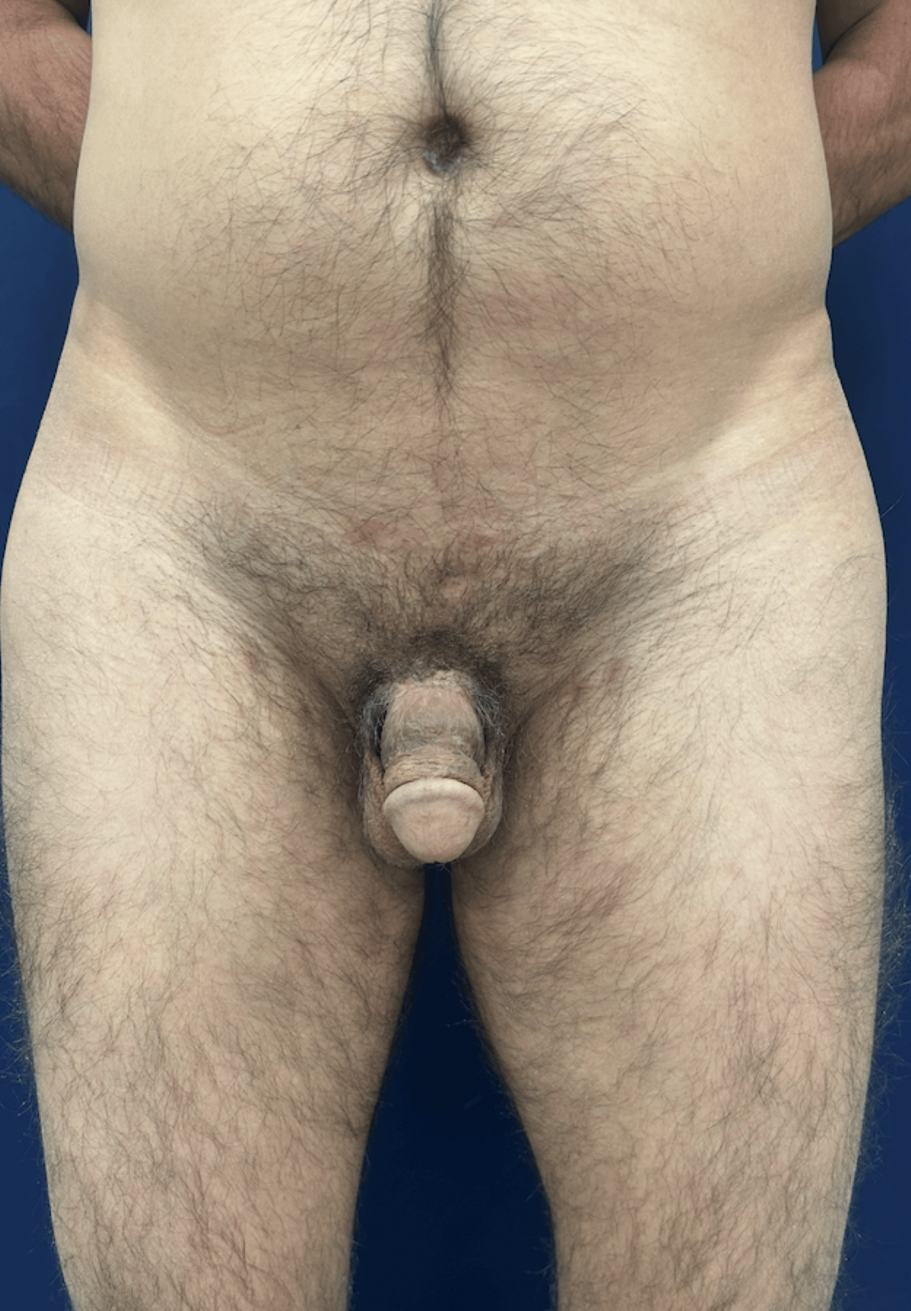
Frontal view of the penis at one-year follow-up, showing preserved morphology and skin integrity.

**Figure 7:**
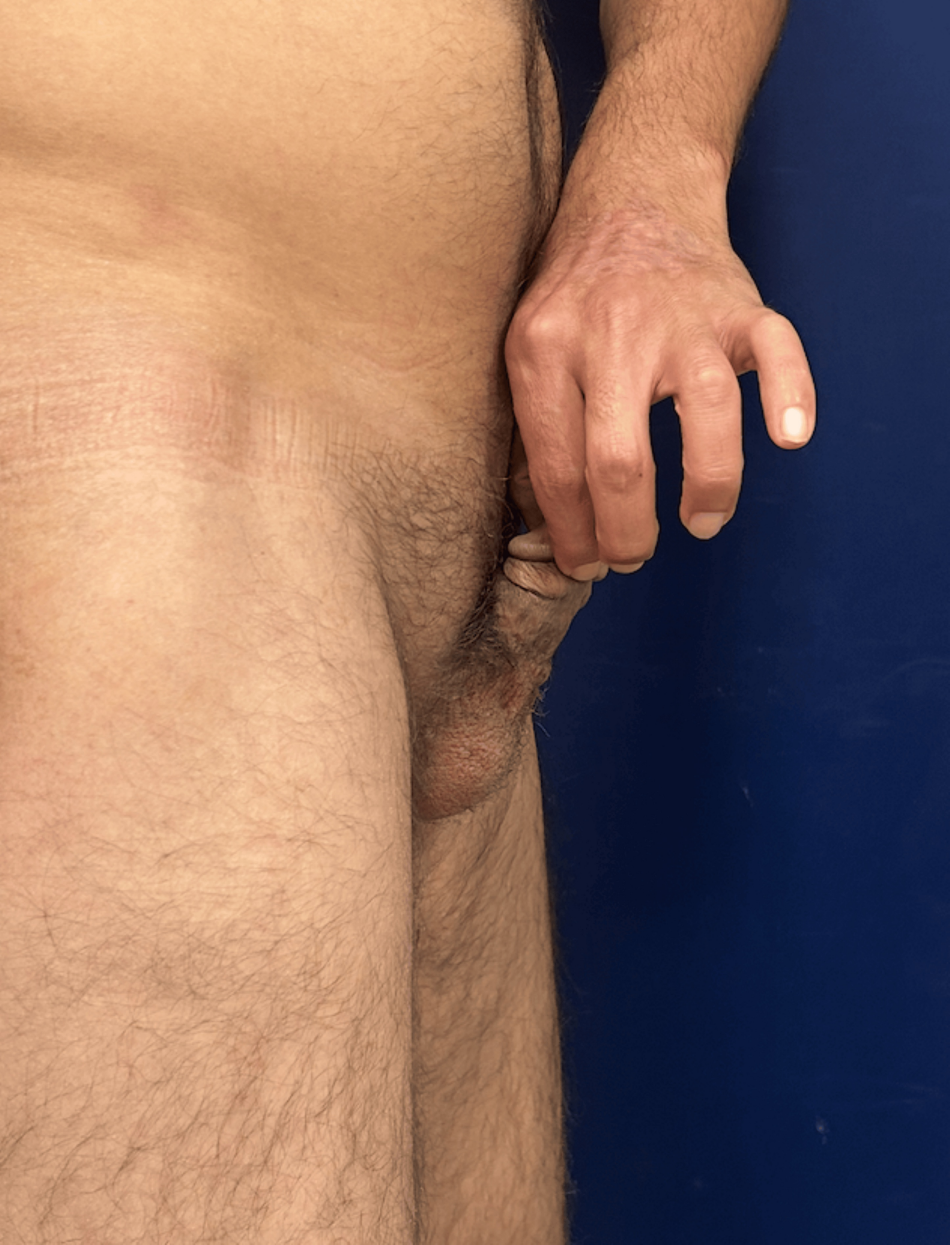
Lateral view of the penis at one-year follow-up, demonstrating preserved length and contour.

At follow-up, the patient was able to void spontaneously with a satisfactory urinary stream. Sensory recovery progressed gradually, with the preservation of pain and light-touch sensation over the penile shaft and glans. No erectile complications related to ischemia or necrosis were observed. The patient remained under regular psychiatric supervision to prevent further self-harm behavior. Photographs at one-year postoperative follow-up demonstrate preserved penile morphology, skin integrity, and satisfactory cosmetic and functional outcomes (Figures 6, 7).

## Discussion

Penile amputation is an uncommon clinical scenario that may result from accidental trauma, circumcision-related injury, interpersonal violence, or deliberate self-harm. Various psychiatric conditions have been implicated in male genital self-mutilation, including disorders within the schizophrenia spectrum, substance abuse, personality disorders, and gender dysphoria [8]. The literature indicates that most cases (approximately 87%) develop during an acute psychotic episode involving self-inflicted injury, with schizophrenia (51%), severe depression (19%), and substance-induced disorders being the most frequently reported underlying causes [9]. Genital self-mutilation in individuals with psychiatric illness is also described as Klingsor syndrome [10], and such patients are known to have a high propensity for repeated self-harming behaviors [7]. This highlights the importance of close psychiatric follow-up and appropriate psychopharmacotherapy in preventing recurrence.

The first known report of penile replantation was published by Ehrich in 1929, involving the realignment of the penile structures without vascular or neural anastomosis [11]. In replantations performed without microvascular repair, tissue viability depends mainly on sinusoidal perfusion within the corpora. However, nonmicrovascular techniques have been associated with a range of complications, including urethral strictures, skin necrosis, sensory loss, fistula formation, and erectile dysfunction [12-15].

Following the description of the first successful microvascular replantation, microsurgical repair has become the preferred approach [4]. Numerous reports have documented successful outcomes after penile autoamputation managed with microvascular techniques. Benefits of microsurgical replantation include improved graft survival, reduced skin loss, better erectile function, and superior cosmetic appearance [12]. Despite a notable incidence of complications such as skin necrosis (54.8%) and venous congestion (20.2%), overall patient satisfaction remains high (91.6%) [2]. Other complications reported in the literature include diminished penile skin sensation, urethral strictures, erectile dysfunction, and urethral fistula formation [16,17]. In our patient, minimal ventral skin necrosis developed, which was debrided and resutured at the first postoperative month, resulting in complete healing. Sensory recovery progressed gradually, and erectile function was preserved.

Attempts to correlate the number of arterial or venous repairs with adequate postoperative perfusion have not yielded definitive conclusions [18]. Wei et al. suggested that at least one dorsal artery should be repaired to ensure sufficient blood supply [19]. Previous studies likewise recommend reconstructing dorsal arteries whenever possible, as the glans receives its entire blood supply from this arterial system [20]. Regarding venous repair, it has been advised to reconstruct as many veins as feasible to minimize postoperative edema and the necrosis of the glans and foreskin. In our case, both the superficial and deep dorsal veins were identified and repaired; however, the dorsal arterial pair was embedded in dense fibrotic tissue, making the arterial anastomosis technically challenging, which is often encountered in secondary amputation cases.

Skin necrosis is the most frequent complication after penile replantation because the proximal penile skin is supplied solely by branches of the external pudendal artery, which can be difficult to identify and repair at the amputation level [20]. Approximately half of the cases demonstrate some degree of skin necrosis, although it is often superficial. When necrosis develops, conservative debridement and resuturation can still provide acceptable outcomes.

The literature on the use of suprapubic catheters during penile replantation is limited; some reports indicate that suprapubic catheterization was not utilized in 82% (9/11) of documented cases [16]. Other studies, however, recommend using both a Foley catheter and a suprapubic catheter for at least one week to reduce urethral anastomotic pressure and thereby lower the risk of urethral stricture. In our case, both a Foley and a suprapubic catheter were used and removed after 30 days. Although the patient regained normal micturition, a mild urethral stricture was noted. While dual catheterization did not entirely prevent stricture formation, the absence of urethral fistula suggests that suprapubic catheter use may help decrease the likelihood of fistula development.

Overall, this case demonstrates the technical feasibility of secondary penile replantation with satisfactory functional and cosmetic outcomes, even in the context of recurrent self-mutilation. Multidisciplinary management, including urologic, microsurgical, and psychiatric care, is crucial to optimize both surgical success and the prevention of recurrence. The rarity of repeated penile amputation emphasizes the clinical significance of this report.

## Conclusions

Penile amputation is associated with profound psychological, functional, and reconstructive consequences. This case demonstrates that secondary penile replantation is technically feasible, and microsurgical repair can achieve penile survival with satisfactory functional and cosmetic outcomes, even after repeated self-amputation. Postoperative complications may occur but can be effectively managed with appropriate surgical care.

The recurrence of self-mutilation in this patient was directly linked to psychiatric relapse, highlighting that successful penile replantation alone is insufficient without comprehensive psychiatric management. Adequate psychopharmacotherapy and long-term psychiatric follow-up are crucial to prevent recurrent autoaggression. Given the rarity of repeated penile amputation with successful replantation, this case supports considering replantation even in secondary cases, provided that a multidisciplinary approach is implemented.
